# 2-Amino-5-chloro­pyridine–fumaric acid (1/2)

**DOI:** 10.1107/S1600536810018192

**Published:** 2010-05-22

**Authors:** Madhukar Hemamalini, Hoong-Kun Fun

**Affiliations:** a X-ray Crystallography Unit, School of Physics, Universiti Sains Malaysia, 11800 USM, Penang, Malaysia

## Abstract

The asymmetric unit of the title compound, C_5_H_5_ClN_2_·0.5C_4_H_4_O_4_, comprises a neutral 2-amino-5-chloro­pyridine mol­ecule and one half of a fumaric acid mol­ecule which lies on an inversion center. The dihedral angle between the pyridine ring and the plane formed by the fumaric acid mol­ecule is 3.22 (8)°. The 2-amino-5-chloro­pyridine mol­ecule is planar, with a maximum deviation of 0.004 (1) Å for the pyridine N atom. In the crystal, the 2-amino-5-chloro­pyridine mol­ecules inter­act with the carboxyl groups of fumaric acid mol­ecules through N—H⋯O and O—H⋯N hydrogen bonds, forming centrosymmetric *R*_2_^2^(8) ring motifs and another N—H⋯O hydrogen bond links these motifs into a two-dimensional network parallel to (100).

## Related literature

For background to the chemistry of substituted pyridines, see: Pozharski *et al.* (1997 ▶); Katritzky *et al.* (1996 ▶). For the details of fumaric acid, see: Batchelor *et al.* (2000 ▶). For details of hydrogen bonding, see: Jeffrey & Saenger (1991 ▶); Jeffrey (1997 ▶); Scheiner (1997 ▶). For hydrogen-bond motifs, see: Bernstein *et al.* (1995 ▶). For bond-length data, see: Allen *et al.* (1987 ▶). For the stability of the temperature controller used in the data collection, see: Cosier & Glazer (1986 ▶).
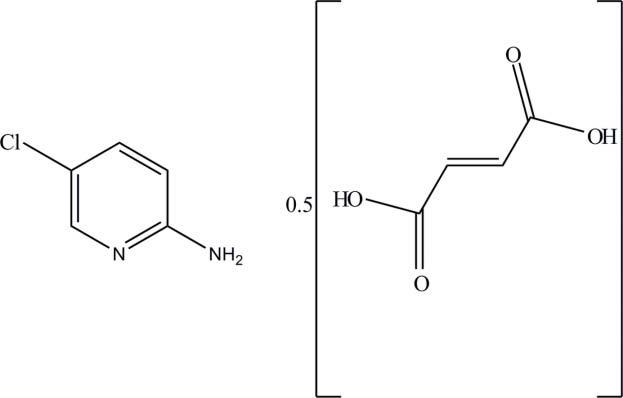


## Experimental

### 

#### Crystal data

C_5_H_5_ClN_2_·0.5C_4_H_4_O_4_
*M* *_r_* = 186.60
Monoclinic, 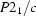

*a* = 13.678 (4) Å
*b* = 5.0586 (15) Å
*c* = 11.531 (3) Å
β = 103.442 (7)°
*V* = 776.0 (4) Å^3^
*Z* = 4
Mo *K*α radiation
μ = 0.45 mm^−1^
*T* = 100 K
0.57 × 0.25 × 0.08 mm

#### Data collection

Bruker APEXII DUO CCD area-detector diffractometer
Absorption correction: multi-scan (*SADABS*; Bruker, 2009 ▶) *T*_min_ = 0.784, *T*_max_ = 0.967
8439 measured reflections
2771 independent reflections
2420 reflections with *I* > 2σ(*I*)
*R*_int_ = 0.036

#### Refinement

*R*[*F* ^2^ > 2σ(*F* ^2^)] = 0.042
*wR*(*F* ^2^) = 0.143
*S* = 1.06
2771 reflections
110 parameters
H-atom parameters constrained
Δρ_max_ = 0.70 e Å^−3^
Δρ_min_ = −0.59 e Å^−3^

### 

Data collection: *APEX2* (Bruker, 2009 ▶); cell refinement: *SAINT* (Bruker, 2009 ▶); data reduction: *SAINT*; program(s) used to solve structure: *SHELXTL* (Sheldrick, 2008 ▶); program(s) used to refine structure: *SHELXTL*; molecular graphics: *SHELXTL*; software used to prepare material for publication: *SHELXTL* and *PLATON* (Spek, 2009 ▶).

## Supplementary Material

Crystal structure: contains datablocks global, I. DOI: 10.1107/S1600536810018192/sj5003sup1.cif

Structure factors: contains datablocks I. DOI: 10.1107/S1600536810018192/sj5003Isup2.hkl

Additional supplementary materials:  crystallographic information; 3D view; checkCIF report

## Figures and Tables

**Table 1:** Hydrogen-bond geometry (Å, °)

*D*—H⋯*A*	*D*—H	H⋯*A*	*D*⋯*A*	*D*—H⋯*A*
O2—H2⋯N1	0.82	1.82	2.5852 (17)	154
N2—H2*A*⋯O1	0.86	2.00	2.856 (2)	171
N2—H2*B*⋯O2^i^	0.86	2.26	3.0718 (18)	158

Symmetry code: (i) 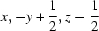.

**Fig. 1.**
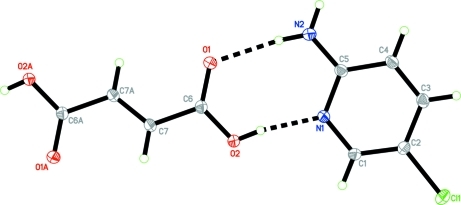
The asymmetric unit of the title compound. Displacement ellipsoids are drawn at the 50% probability level.

**Fig. 2.**
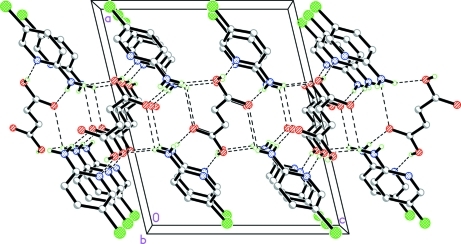
The crystal packing of the title compound, showing hydrogen-bonded (dashed lines) networks. H atoms not involved in hydrogen bond interactions are omitted for clarity.

## supplementary crystallographic information

### Comment

Pyridine and its derivatives play an important role in heterocyclic chemistry (Pozharski *et al.*, 1997; Katritzky *et al.*, 1996). They are often involved in hydrogen-bond interactions (Jeffrey & Saenger, 1991; Jeffrey, 1997; Scheiner, 1997). Fumaric acid is among the organic compounds widely found in nature, and is key intermediate in the biosynthesis of organic acids. Fumaric acid is of interest since it is known to form supramolecular assemblies with *N*–aromatic complexes (Batchelor *et al.*, 2000). In order to study some interesting hydrogen bonding interactions, the synthesis and structure of the title compound, (I), is presented here.

The asymmetric unit of the title compond consists of a 2-amino-5- chloropyridine molecule and a half of the fumaric acid molecule (Fig. 1). The planar fumaric acid molecule is  centrosymmetric with the mid-point of the C═C double bond located at an inversion center. The C6–O1 bond distance of 1.2375 (17) Å is much shorter than the C6–O2 bond distance of 13061 (16) Å, suggesting that the carboxyl group is not deprotonated in the crystal structure. The 2-amino- 5-chloropyridine molecule is planar, with a maximum deviation of 0.004 (1) Å for atom N1.  The bond lengths (Allen *et al.*, 1987) and angles are normal.

In the crystal packing (Fig. 2), the 2-amino-5-chloropyridine molecules interact with the carboxyl groups (O1 & O2) of fumaric acid molecules through N2—H2A···O1 and O2—H2···N1 hydrogen bonds (Table 1), forming cyclic hydrogen-bonded motifs *R*_2_^2^(8) (Bernstein *et al.*, 1995) and the N2—H2B···O2 hydrogen bond links these motifs into a two-dimensional network parallel to (100) plane.

### Experimental

A hot methanol solution (20 ml) of 2-amino-5-chloropyridine (64 mg, Aldrich) and fumaric acid (58 mg, Merck) was mixed and warmed over a a magnetic stirrer hotplate for a few minutes. The resulting solution was allowed to cool slowly at room temperature and crystals of the title compound appeared after a few days.

### Refinement

All hydrogen atoms were positioned geometrically [C–H = 0.93 Å, N–H = 0.86 Å and O–H = 0.82 Å] and were refined using a riding model, with U_iso_(H) = 1.2 U_eq_(C, N) or 1.5 U_eq_(O).

### Crystal data

**Table:**

C_5_H_5_ClN_2_·0.5C_4_H_4_O_4_	*F*(000) = 384
*M**_r_* = 186.60	*D*_x_ = 1.597 Mg m^−^^3^
Monoclinic, *P*2_1_/*c*	Mo *K*α radiation, λ = 0.71073 Å
Hall symbol: -P 2ybc	Cell parameters from 3958 reflections
*a* = 13.678 (4) Å	θ = 3.6–36.5°
*b* = 5.0586 (15) Å	µ = 0.45 mm^−^^1^
*c* = 11.531 (3) Å	*T* = 100 K
β = 103.442 (7)°	Plate, colourless
*V* = 776.0 (4) Å^3^	0.57 × 0.25 × 0.08 mm
*Z* = 4	

### Data collection

**Table:**

Bruker APEXII DUO CCD area-detector diffractometer	2771 independent reflections
Radiation source: fine-focus sealed tube	2420 reflections with *I* > 2σ(*I*)
graphite	*R*_int_ = 0.036
φ and ω scans	θ_max_ = 32.5°, θ_min_ = 3.6°
Absorption correction: multi-scan (*SADABS*; Bruker, 2009)	*h* = −20→16
*T*_min_ = 0.784, *T*_max_ = 0.967	*k* = −7→7
8439 measured reflections	*l* = −17→17

### Refinement

**Table:**

Refinement on *F*^2^	Primary atom site location: structure-invariant direct methods
Least-squares matrix: full	Secondary atom site location: difference Fourier map
*R*[*F*^2^ > 2σ(*F*^2^)] = 0.042	Hydrogen site location: inferred from neighbouring sites
*wR*(*F*^2^) = 0.143	H-atom parameters constrained
*S* = 1.06	*w* = 1/[σ^2^(*F*_o_^2^) + (0.0898*P*)^2^ + 0.4122*P*] where *P* = (*F*_o_^2^ + 2*F*_c_^2^)/3
2771 reflections	(Δ/σ)_max_ = 0.001
110 parameters	Δρ_max_ = 0.70 e Å^−^^3^
0 restraints	Δρ_min_ = −0.59 e Å^−^^3^

### Special details

**Table:**

Experimental. The crystal was placed in the cold stream of an Oxford Cryosystems Cobra open-flow nitrogen cryostat (Cosier & Glazer, 1986) operating at 100.0 (1) K.
Geometry. All s.u.'s (except the s.u. in the dihedral angle between two l.s. planes) are estimated using the full covariance matrix. The cell s.u.'s are taken into account individually in the estimation of s.u.'s in distances, angles and torsion angles; correlations between s.u.'s in cell parameters are only used when they are defined by crystal symmetry. An approximate (isotropic) treatment of cell s.u.'s is used for estimating s.u.'s involving l.s. planes.
Refinement. Refinement of F^2^ against ALL reflections. The weighted R-factor wR and goodness of fit S are based on F^2^, conventional R-factors R are based on F, with F set to zero for negative F^2^. The threshold expression of F^2^ > 2σ(F^2^) is used only for calculating R-factors(gt) etc. and is not relevant to the choice of reflections for refinement. R-factors based on F^2^ are statistically about twice as large as those based on F, and R- factors based on ALL data will be even larger.

### Fractional atomic coordinates and isotropic or equivalent isotropic displacement parameters (Å^2^)

**Table:**

	*x*	*y*	*z*	*U*_iso_*/*U*_eq_	
Cl1	0.03621 (3)	−0.27603 (7)	0.40770 (3)	0.01778 (13)	
N1	0.25244 (9)	0.1952 (2)	0.34964 (11)	0.0130 (2)	
N2	0.34626 (11)	0.2094 (3)	0.20667 (12)	0.0182 (3)	
H2A	0.3772	0.3457	0.2420	0.022*	
H2B	0.3615	0.1481	0.1436	0.022*	
C1	0.17992 (10)	0.0834 (3)	0.39610 (12)	0.0133 (2)	
H1A	0.1661	0.1550	0.4648	0.016*	
C2	0.12664 (10)	−0.1319 (3)	0.34452 (12)	0.0133 (2)	
C3	0.14697 (11)	−0.2407 (3)	0.24014 (13)	0.0149 (3)	
H3A	0.1109	−0.3861	0.2037	0.018*	
C4	0.22039 (11)	−0.1301 (3)	0.19295 (12)	0.0153 (3)	
H4A	0.2349	−0.2000	0.1242	0.018*	
C5	0.27436 (10)	0.0926 (3)	0.24997 (12)	0.0133 (2)	
O1	0.44882 (8)	0.6317 (2)	0.34908 (10)	0.0177 (2)	
O2	0.34481 (8)	0.5851 (2)	0.47334 (9)	0.0143 (2)	
H2	0.3331	0.4402	0.4412	0.022*	
C6	0.41907 (10)	0.6973 (3)	0.43840 (12)	0.0127 (2)	
C7	0.46749 (10)	0.9183 (3)	0.51670 (12)	0.0138 (2)	
H7A	0.4520	0.9427	0.5904	0.017*	

### Atomic displacement parameters (Å^2^)

**Table:**

	*U*^11^	*U*^22^	*U*^33^	*U*^12^	*U*^13^	*U*^23^
Cl1	0.01527 (19)	0.01894 (19)	0.0195 (2)	−0.00319 (10)	0.00484 (13)	0.00275 (11)
N1	0.0146 (5)	0.0132 (5)	0.0113 (5)	−0.0018 (4)	0.0033 (4)	−0.0012 (4)
N2	0.0230 (6)	0.0172 (6)	0.0176 (6)	−0.0049 (4)	0.0111 (5)	−0.0033 (4)
C1	0.0145 (6)	0.0146 (5)	0.0112 (5)	−0.0013 (4)	0.0036 (4)	−0.0002 (4)
C2	0.0125 (5)	0.0142 (6)	0.0127 (5)	−0.0013 (4)	0.0021 (4)	0.0015 (4)
C3	0.0157 (6)	0.0144 (5)	0.0133 (6)	−0.0021 (4)	0.0008 (5)	−0.0012 (4)
C4	0.0189 (6)	0.0148 (6)	0.0121 (5)	−0.0006 (4)	0.0035 (4)	−0.0020 (4)
C5	0.0157 (6)	0.0131 (5)	0.0109 (5)	0.0001 (4)	0.0029 (4)	−0.0004 (4)
O1	0.0197 (5)	0.0184 (5)	0.0175 (5)	−0.0051 (4)	0.0089 (4)	−0.0060 (4)
O2	0.0155 (5)	0.0153 (4)	0.0130 (4)	−0.0039 (3)	0.0049 (4)	−0.0023 (3)
C6	0.0120 (5)	0.0124 (5)	0.0133 (6)	−0.0001 (4)	0.0021 (4)	−0.0007 (4)
C7	0.0144 (6)	0.0135 (5)	0.0133 (6)	−0.0011 (4)	0.0030 (4)	−0.0028 (4)

### Geometric parameters (Å, °)

**Table:**

Cl1—C2	1.7352 (14)	C3—H3A	0.9300
N1—C1	1.3555 (17)	C4—C5	1.422 (2)
N1—C5	1.3565 (17)	C4—H4A	0.9300
N2—C5	1.3393 (18)	O1—C6	1.2375 (17)
N2—H2A	0.8600	O2—C6	1.3061 (16)
N2—H2B	0.8600	O2—H2	0.8200
C1—C2	1.3675 (19)	C6—C7	1.4920 (19)
C1—H1A	0.9300	C7—C7^i^	1.335 (3)
C2—C3	1.409 (2)	C7—H7A	0.9300
C3—C4	1.368 (2)		
			
C1—N1—C5	120.10 (12)	C3—C4—C5	119.37 (13)
C5—N2—H2A	120.0	C3—C4—H4A	120.3
C5—N2—H2B	120.0	C5—C4—H4A	120.3
H2A—N2—H2B	120.0	N2—C5—N1	118.25 (13)
N1—C1—C2	121.69 (12)	N2—C5—C4	121.64 (13)
N1—C1—H1A	119.2	N1—C5—C4	120.10 (12)
C2—C1—H1A	119.2	C6—O2—H2	109.5
C1—C2—C3	119.45 (12)	O1—C6—O2	124.75 (13)
C1—C2—Cl1	120.79 (11)	O1—C6—C7	121.27 (12)
C3—C2—Cl1	119.76 (11)	O2—C6—C7	113.98 (12)
C4—C3—C2	119.29 (13)	C7^i^—C7—C6	121.41 (16)
C4—C3—H3A	120.4	C7^i^—C7—H7A	119.3
C2—C3—H3A	120.4	C6—C7—H7A	119.3
			
C5—N1—C1—C2	−0.5 (2)	C1—N1—C5—N2	179.77 (13)
N1—C1—C2—C3	−0.3 (2)	C1—N1—C5—C4	0.9 (2)
N1—C1—C2—Cl1	178.49 (11)	C3—C4—C5—N2	−179.38 (14)
C1—C2—C3—C4	0.6 (2)	C3—C4—C5—N1	−0.5 (2)
Cl1—C2—C3—C4	−178.16 (11)	O1—C6—C7—C7^i^	11.5 (3)
C2—C3—C4—C5	−0.2 (2)	O2—C6—C7—C7^i^	−168.52 (17)

Symmetry codes: (i) −*x*+1, −*y*+2, −*z*+1.

### Hydrogen-bond geometry (Å, °)

**Table:**

*D*—H···*A*	*D*—H	H···*A*	*D*···*A*	*D*—H···*A*
O2—H2···N1	0.82	1.82	2.5852 (17)	154
N2—H2A···O1	0.86	2.00	2.856 (2)	171
N2—H2B···O2^ii^	0.86	2.26	3.0718 (18)	158

Symmetry codes: (ii) *x*, −*y*+1/2, *z*−1/2.
